# SARS-CoV-2 Main Protease Active Site Ligands in the Human Metabolome

**DOI:** 10.3390/molecules26051409

**Published:** 2021-03-05

**Authors:** Anna Maria Sardanelli, Camilla Isgrò, Luigi Leonardo Palese

**Affiliations:** 1 Department of Basic Medical Sciences, Neurosciences and Sense Organs, University of Bari “Aldo Moro”, Piazza G. Cesare 11, 70124 Bari, Italy; camilla.isgro@uniba.it; 2 Department of Medicine, University Campus Bio-Medico of Rome, Via Alvaro del Portillo 21, 00128 Rome, Italy

**Keywords:** coronavirus, COVID-19, SARS-CoV-2, M^pro^, 3C-like protease, 3CL protease, protease inhibitors, metabolome, silybin, silymarin

## Abstract

In late 2019, a global pandemic occurred. The causative agent was identified as a member of the *Coronaviridae* family, called severe acute respiratory syndrome coronavirus 2 (SARS-CoV-2). In this study, we present an analysis on the substances identified in the human metabolome capable of binding the active site of the SARS-CoV-2 main protease (M^pro^). The substances present in the human metabolome have both endogenous and exogenous origins. The aim of this research was to find molecules whose biochemical and toxicological profile was known that could be the starting point for the development of antiviral therapies. Our analysis revealed numerous metabolites—including xenobiotics—that bind this protease, which are essential to the lifecycle of the virus. Among these substances, silybin, a flavolignan compound and the main active component of silymarin, is particularly noteworthy. Silymarin is a standardized extract of milk thistle, *Silybum marianum*, and has been shown to exhibit antioxidant, hepatoprotective, antineoplastic, and antiviral activities. Our results—obtained in silico and in vitro—prove that silybin and silymarin, respectively, are able to inhibit M^pro^, representing a possible food-derived natural compound that is useful as a therapeutic strategy against COVID-19.

## 1. Introduction

In 2019, an outbreak was reported in China [1], rapidly resulting in a pandemic named coronavirus disease 2019 (COVID-19) [2]. Severe acute respiratory syndrome coronavirus 2 (SARS-CoV-2) has been identified as the causative agent of this pathology, a new betacoronavirus [3], which is, thus, added to other coronaviruses (CoVs) implicated in different infectious diseases in humans, namely strains 229E, NL63, OC43, HKU1, Middle East Respiratory Syndrome (MERS-CoV), and severe acute respiratory syndrome coronavirus (SARS-CoV). Although vaccines are now available, the large-scale immunization of the entire world population will require a considerable amount of time. Furthermore, the actual emergence of new variants of SARS-CoV-2 [4,5,6,7,8,9], as well as the possibility that other CoVs may emerge as pathogens in the future, make the search for drugs/remedies against this virus important.

CoVs have large, single-stranded, positive-sense RNA genomes [10,11,12], particularly encoding the spike (S), membrane (M), envelop (E), and nucleocapsid (N) proteins. Two-thirds of the RNA genome is covered by ORF1a and ORF1b, which produce two polyproteins, PP1a and PP1ab, and two cysteine proteases involved in the specific cuts of these polyproteins [13,14,15,16,17,18]. The first is a papain-like protease (PLpro), which performs three cleavage reactions, while the other CoV protease is a chymotrypsin-like enzyme, known as main protease (M^pro^) or 3C-like protease (3CLpro) because of its similarity to the picornavirus 3C protease. M^pro^ is responsible for 11 cuts. M^pro^ recognition sequence is X-(L/F/M)-Q | (G/A/S)-X (X: any amino acid; |: cleavage site). These proteases are therefore excellent targets for the development of a specific antiviral therapy, and specific inhibitors of M^pro^ are in the early stages of clinical development [19,20]. Development of new drugs generally requires several years and high costs. Hence, the need to identify already approved drugs effective against SARS-CoV-2. Drug repurposing efforts have led to a large number of candidates using different approaches [21], which are interesting, not only for the research of drugs of immediate use, but also for identifying new avenues for future developments. We therefore used as a starting point for this analysis the molecules, both of endogenous and xenobiotic origin, identified in the human metabolome. In this work, we describe the results of our approach for the identification of molecules able to bind the active site of M^pro^ and, therefore, act as inhibitors of this enzyme.

Among these substances, silybin is particularly noteworthy. Silybin, also known as flavobin, belongs to a class of organic compounds known as flavonolignans, non-conventional lignans derived from flavonoids. They are characterized by a p-dioxin ring substituted at one carbon atom by a C3C6 (phenylpropan) group and fused to the B-ring of the 2-phenylchromene moiety [22]. Silybin is the main active component of silymarin, extracted from milk thistle, *Silybum marianum* [23]. This food-derived natural compound contains approximately 70% to 80% of the silymarin flavonolignans (silymarin complex) and approximately 20% to 30% of a chemically undefined fraction, comprising polymeric and oxidized polyphenolic compounds known as polyphenolic fraction (PP). Besides silybin (C25H22O10, PubChem CID: 31553), which is a mixture of diastereomers A and B in, approximately, a 1:1 proportion, considerable amounts of other flavonolignans are present in the silymarin complex, namely isosilybin, dehydrosilybin, silychristin, silydianin, and a few flavonoids, mainly taxifolin [23].

Silymarin is already well known for its hepatoprotective functions and a growing body of evidences suggests that this compound has inhibitory activity against numerous viruses, belonging to different viral families [24].

However, there is very poor evidence about an antiviral effect of silymarin and its derivatives on the *Coronaviridae* family [25].

In this study, we aimed to identify some molecules of various origins, present in the human metabolome, which could be effective against the SARS-CoV-2 M^pro^. Through a series of in silico and in vitro analyses, we have identified silymarin, as a promising natural, food-derived antiviral molecule that can inhibit the aforementioned enzyme activity, representing a possible starting point for the development of effective antiviral therapies.

## 2. Results

### 2.1. The Docking Target

**Figure 1:**
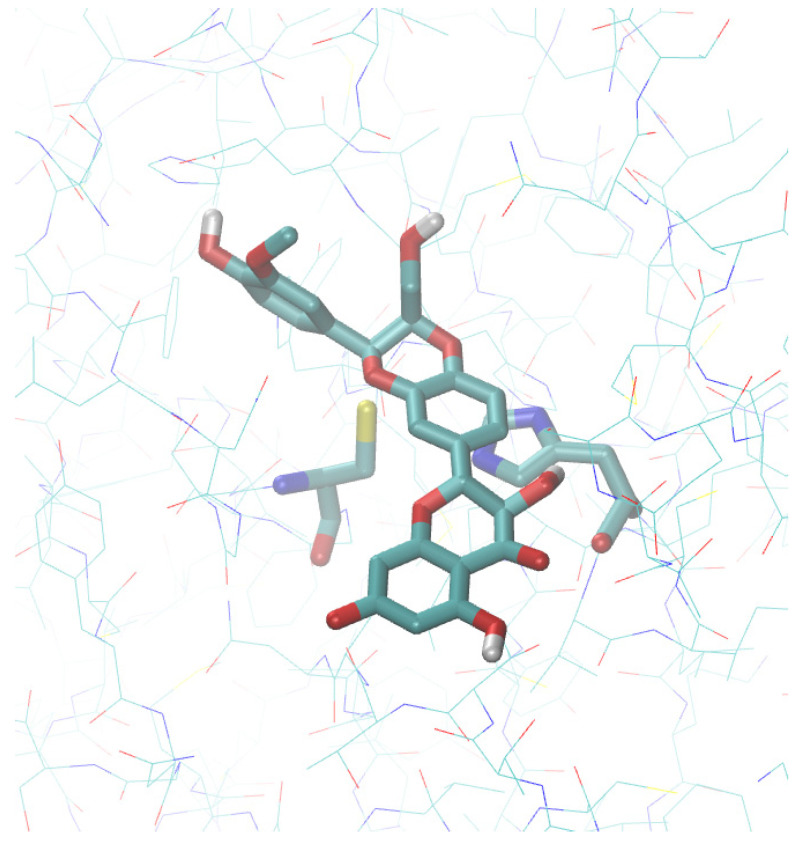
Molecular docking of silybin at the active site of severe acute respiratory syndrome coronavirus 2 (SARS-COV-2) M^pro^. The protein structure corresponds to the PDB entry 5RET.

The M^pro^ structure is remarkably similar in all CoVs [13]. The enzyme is a homodimer, and each monomer consists of two domains: I (residues 8-101 in 6LU7 [26]) and II (residues 102-184). The α-helical domain (domain III) containing residues 201-303 is involved in the dimerization. The active site of M^pro^ comprises a catalytic dyad consisting of the conserved residues, His-41 and Cys-145, which operate a general base catalysis mechanism [27]. An interesting feature from a biochemical point of view is that a water molecule at 3.2–3.3 Å from the N-є of His-41 is visible in all of the structures of this enzyme reported in the Protein Data Bank (PDB); thus, suggesting that a catalytic triad is at work in SARS-CoV-2 M^pro^ [28]. A detailed analysis of these structures has already been reported elsewhere and will not be repeated in detail here [28]. We recall only that the M^pro^ structures are grouped in a single cluster from which some outliers detach along the first principal component. Based on these analyses, we chose 5RET [29] as representative of the centroid of the distribution. After ligand removal from the active site, this structure was exploited to obtain the receptor for docking, as described in the Materials and Methods section (Figure 1).

### 2.2. The Ligand Database

**Figure 2:**
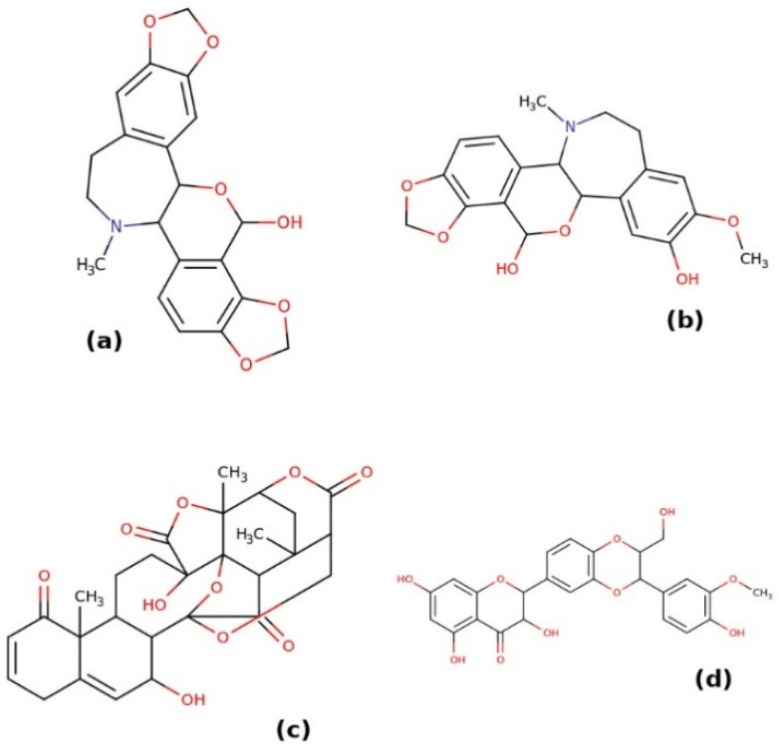
Structures of the topmost metabolites able to bind the M^pro^ active site. These compounds are isorheagenine (**a**), n-methyl-14-o-demethylepiporphyroxine (**b**), physalin N (**c**), and silybin (**d**).

The strategy used in this work—to identify substances with a known toxicological profile to be used as drug candidates in the therapy of COVID-19—was to evaluate the ability of different substances present in the human metabolome to inhibit SARS-CoV-2 M^pro^. We implemented this strategy because endogenous metabolites and a large number of xenobiotics are present in the human metabolome. We first obtained all of the molecules that met the criteria for drug-like molecules present in the HMDB (see Materials and Methods section). Among these criteria, the molecular weight was particularly important, which had to be between 250 and 1000 Da. This was based on the empirical observation that most substances with pharmacological activity fall in this molecular weight range. The HMBD entries considered at this stage are reported in the file HMDB_drug_like_subset.csv (see Supplementary data). Using the SMILES codes of these substances, we searched the ZINC database for metabolites, for which it was possible to obtain an adequate *pdbqt* file for molecular docking. It should be noted that, in some cases, the research on ZINC carried out in this way does not exactly return the molecule reported in HMDB, particularly if different isomers of the molecule are present. In any case, these are closely related molecules and expected in the human metabolome. Our final dataset included 995 ZINC entries (see the zinc_id.csv file in Supplementary data) on which subsequent analyses were performed. Docking was carried out on the 5RET structure of the M^pro^. The results of this analysis are reported in the supplementary.csv file. The observed binding affinities of these substances to M^pro^ range from −4.3 to −9.3 kcal mol^−1^. Many of these are actually poorly studied from a pharmacological point of view or even not purchasable. Substances with best scores in the in silico set up are reported in Figure 2. Based on the docking analysis, the substance with the highest binding affinity to the active site of M^pro^ is isorheagenine (ZINC253387561, HMDB0029360), with a calculated binding affinity of −9.3 kcal mol^−1^. This is an alkaloid identified in *Papaver rhoeas*, but of which there are no pharmacological and toxicological studies [30]. Very similar in structure to isorheagenine is the compound n-methyl-14-o-demethylepiporphyroxine (ZINC100772908, HMDB0030171), which shows a binding affinity equal to −8.9/−8.8 kcal mol^−1^, but also for this compound, there are no pharmacological and toxicological data in the literature. Likewise, there are no such studies on physalin N (HMDB0039080), an atypical steroid described in plants of the genus *Physalis*. Several stereoisomers of this substance are reported in ZINC, but only some of these precomputed structural files for molecular docking are available (ZINC100782941, ZINC100782938, ZINC100782935, ZINC96273223). The best result was obtained with ZINC100782935, for which a pose with a binding affinity of −9.2 kcal mol^−1^ was observed, while with the other stereoisomers, poses were obtained with binding affinity values between −8.1 and −8.9 kcal mol^−1^. Due to the lack of information in the literature on its toxicity and pharmacological action, this compound was not considered further in this work.

**Figure 3:**
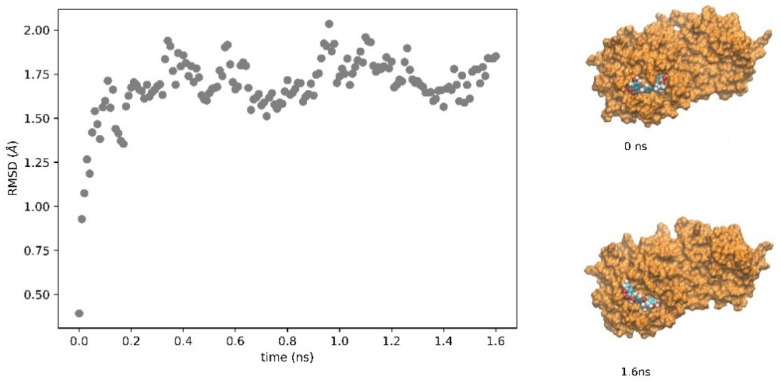
RMSD of the M^pro^ linked to silybin. The SARS-CoV2 protease was subjected to molecular dynamics in a water box with periodic boundary conditions, as described in the Methods section, to evaluate the stability of the protease-silybin (ZINC2033588) complex. On the right in the figure, the conformations of the protease–ligand complex at time zero (start of the simulation after minimization and equilibration, structure at the top right) and at 1.6 ns (structure at the bottom right) are shown. The protease is reported as the orange surface and silybin as the van der Waals structure.

A very interesting compound, among the best candidates in the metabolome as potential inhibitors of M^pro^, is ZINC2033588, one of the stereoisomers obtained from the query on ZINC using the SMILES code of HMDB0030583, whose common name is silybin. This compound exhibits a binding affinity for the M^pro^ active site of −8.9 kcal mol^−1^. What makes this compound particularly interesting, on which the subsequent analysis has focused (see below), is that it is a well-known and widely used compound, belonging to the flavonolignan family, and is the main active component of the aforementioned silymarin. As this compound was further analyzed in vitro (see below), the stability of the complex obtained by molecular docking was analyzed by a molecular dynamics analysis (see Methods). As shown in Figure 3, after equilibration and reaching a steady state (judged by the protein’s root-mean-square deviation (RMSD), the complex is stable on the binding site on a nanosecond timescale, with a distance between the cysteine mass centers of the active site and that of the ligand equal to 8.4 ± 0.7 Å (means ± sd).

Finally, our in silico analyses revealed a large number of high affinity compounds, including rubroskyrin, cepagenin, and a number of compounds conjugated with glucuronic acid (4-hydroxyphenytoin glucuronide, kaempferol 3-glucuronide, 11-oxo-androsterone glucuronide), which have not been further evaluated, because toxic, not described from a pharmacological point of view, or were not available on the market.

### 2.3. Effect of Silymarin on SARS-CoV-2 M^pro^ Activity

**Figure 4:**
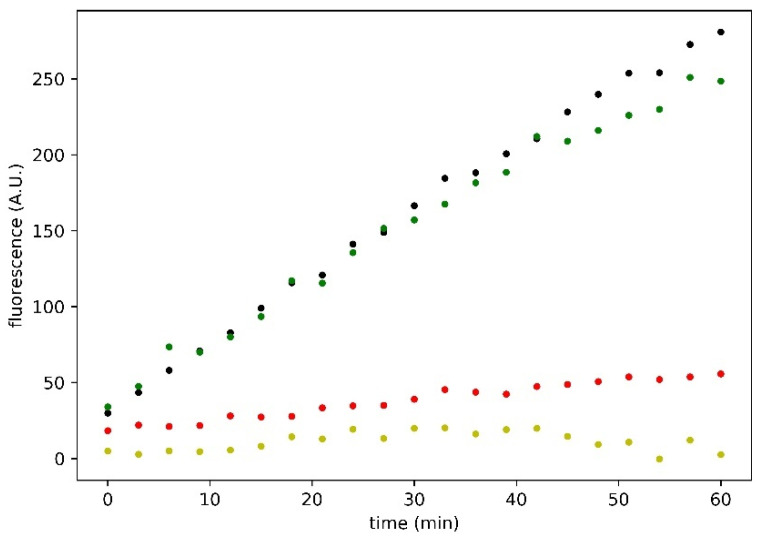
Time course of SARS-CoV-2 M^pro^ enzymatic activity in response to inhibitory compounds. Black, green, red, and yellow dots represent control, 100 µM taxifolin, 95.6 µM silybin A + silybin B, and 100 µM GC376, respectively. Reported traces are the average of three experiments.

The efficacy of silymarin in inhibiting the SARS-CoV-2 M^pro^ was tested by in vitro assays to validate the evidences acquired in silico for silybin, the main component of silymarin. These analyses were performed using the purified enzyme as described in the Materials and Methods section. The enzymatic kinetic was evaluated at concentrations of enzyme and substrate, such as to have linear kinetics (zero-order kinetics). The measurements of the enzymatic V_0_ at various concentrations of silymarin (expressed as silybin A + silybin B, considering their *w*/*w* fraction in silymarin, as reported in [23]), were assessed at least in triplicate. Figure 4 shows an example of the traces obtained in an experimental session. The residual activity of the inhibited enzyme by silymarin was calculated as the ratio between the V_0_ of the enzyme and the V_0_ of the latter in the absence of silymarin.

We also tested the single separate effect of taxifolin, a flavonolignan present in the silymarin complex, as shown in Figure 4, but it does not seem to exercise any significant inhibitory effect on the M^pro^, as shown by the residual enzyme activity (86%), compared to the control M^pro^ activity in the absence of any substances. We also performed our experiments in the presence of 1 mM dithiothreitol (DTT). We observed that the V_0_ of the enzyme in the presence (and the absence) of DTT was 3.58 A.U. min^−1^ (S.E.M. ± 0.09) and 4.35 A.U. min^−1^ (S.E.M. ± 0.12), respectively. Moreover, the silymarin inhibitory action decreases in the presence of 1 mM DTT and the residual activity is around 53.9% (S.E.M. ± 0.9) (data not shown). In the absence of DTT, the residual M^pro^ activity is around 27.7% at 100 µM of silymarin (S.E.M. ± 1.9), with respect to the control. To date, GC376, an experimental veterinary drug (IC50 equal to 0.42 µM) is the best term of comparison that can be used in evaluating the M^pro^ inhibition activity of SARS-CoV-2, so this drug represents the positive control in our experimental sessions.

**Figure 5:**
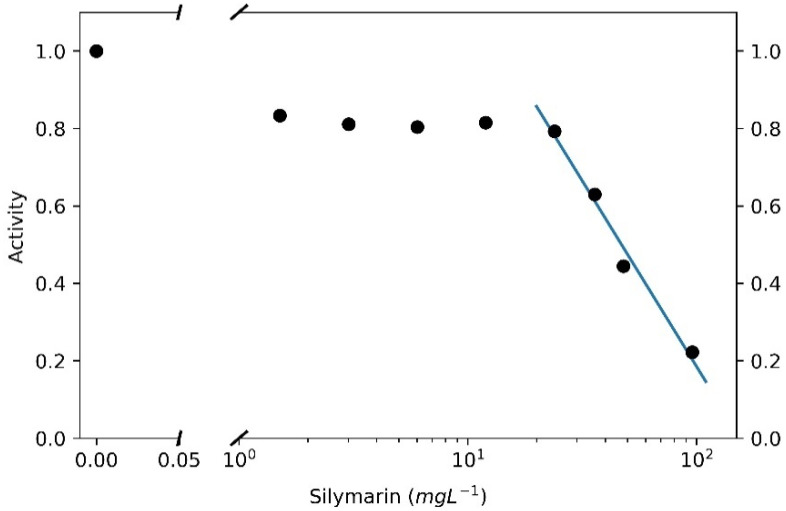
Inhibition of M^pro^ by silymarin. Circles represent the mean of at least three independent replicates.

Using the data obtained in the experimental DTT free conditions, the IC50 of the silymarin was calculated. The estimation of the IC50 obtained by considering a linear function in the semi-logarithmic graph is equal to 47.11 mg L^−1^, as shown in Figure 5. This value, considering the calculated mean content of silybin A and silybin B in the standardized silymarin extract, as reported in [23], is equal to 46.88 µM. This value, in order of micromolar concentration, is really interesting, and indicates that silymarin can be considered one of the most effective M^pro^ inhibitors among the potential natural food-derived compounds capable of inhibiting SARS-CoV-2 (compare the trace shown in Figure 4, obtained at almost the same concentration value of 100 µM GC376).

## 3. Discussion

The xenobiotics can be either drugs or substances deriving from their transformation and conjugation by the detoxification systems, and substances of natural origin, particularly secondary metabolites of vegetable origin. These can result from the ingestion of food or from the deliberate intake of particular preparations (i.e., herbal products). Although these substances are very often present in minimal quantities in the analyzed metabolomes, nevertheless their pharmacological characteristics, and their toxicity, have been extensively described in many cases. It is therefore an innovative strategy, although similar in some respects to traditional “drug repurposing”, to investigate their effects on SARS-CoV-2 M^pro^. Moreover, although this is only a hypothesis at this stage, it is possible that, at least in part, the considerable individual variability in the clinical outcomes of SARS-CoV-2 infections may be directly attributable to individual metabolome differences.

Although the main target of this analysis was the search for xenobiotic substances contained in the human metabolome, some endogenous metabolites deserve a separate mention that could be interesting to analyze in future studies. We particularly remember dTDP-4-dehydro-6-deoxy-L-mannose and NADPH, the reduced form of the nicotinamide adenine dinucleotide phosphate. Both metabolites show a significant interaction in silico with the active site of M^pro^, with a binding energy of −8.5 and −8.6 kcal mol^−1^, respectively, as reported in the supplementary.csv file.

According to our results, in a previous in silico study the authors showed that several selected structures, such as NAD-NADH or NAD-like derivatives, present the best fit in the SARS CoV-2 M^pro^ cavity, suggesting a pivotal role of these molecules in the modulation of SARS CoV-2 infection in case of chronic oxidative stress at a cellular level [31]. Moreover, biliverdin was significant in silico affinity for the M^pro^ active site, with a binding energy of −8.4 kcal mol^−1^.

In recent years (2020, in particular), several studies have focused on the research of natural food-derived compounds exhibiting antiviral activities both in silico and in vitro [32,33,34]. Among these substances, flavonoids are particularly noteworthy [35,36,37]. One of the first papers exploring the antiviral effects of flavonoids on coronaviruses was conducted in 1990 [38]. Here, the authors showed that quercetin, at a concentration value of 60 μg/mL, reduced infectivity of human and bovine coronaviruses, OC43, and NCDCV by 50%. Quercetin may be considered a promising candidate for further preclinical studies as its ability to influence the thermal stability of SARS-CoV-2 M^pro^, interact with SARS-CoV-2 M^pro^, and bind to its active site has recently been demonstrated [39]. Based on the results obtained in silico, our group decided to test, by a series of in vitro experiments, the effect of a natural compound known as silymarin on SARS-CoV-2 M^pro^. Silymarin exerts a remarkable inhibitory action, as the EC50 observed by our research group is in the micromolar range. In addition, an interesting parameter is the residual activity of the M^pro^ because of its very low value. We also analyzed the potential effect of taxifolin, a component of the silymarin complex. Docking has shown that taxifolin is not an excellent protease ligand (calculated binding energy −7.7 kcal mol^−1^) and this was further confirmed by the experimental analysis (see Figure 4). These data confirm our hypothesis that the active component of silymarin is silybin.

The choice of using the silymarin complex and not silybin (investigated in silico) is because it is more readily accessible to clinicians and patients, because it is commercially available in the form of supplements containing 51–78% *w*/*w* of silymarin.

However, a study conducted using computational and experimental approaches has delineated the ability of silybin to target the virus replication machinery by targeting RdRp/nsp12, a central component of a multi-subunit RNA-synthesis complex [25]. Silymarin, and its derivative silybin, present another interesting property as reactive oxygen species (ROS) scavengers and modulators of glutathione levels in various organs [40]. Thus, despite our analysis showed that the silymarin inhibitory action decreases in the presence of DTT, its efficacy may not be reduced in cells or tissues containing high concentrations of glutathione.

Finally, pharmacokinetic studies [41] have shown that silymarin is absorbed by the oral route and distributes into the alimentary tract. It is subject to enterohepatic circulation, ensuring that low doses of intake could be sufficient. Acute, subacute, and chronic toxicity is very low. Silymarin can also be consumed in pregnancy because it is devoid of embryotoxic potential [41]. Moreover, silymarin is safe at therapeutic doses and is well tolerated at high doses [42]. For these reasons, we hypothesize that it can be used not only as a therapeutic strategy, but also as a preventive measure against SARS-CoV-2 infection, because of a possible maintenance of its circulating levels. Surely, to confirm this hypothesis, future clinical trials are needed.

In conclusion, our study proves that silymarin, as a natural food-derived compound, whose pharmacological, toxicological, and therapeutic profiles are known, can be considered a promising and safe therapeutic strategy against COVID-19. Obviously, these data obtained in silico and in vitro should be confirmed by further in vivo studies, to set the optimal dosages, and assess the efficacy of this compound in inhibiting SARS-CoV-2 M^pro^ in humans.

## 4. Materials and Methods

### 4.1. In Silico Analyses

The human metabolome is the complete collection of molecules present in the human body, including amino acids, peptides, lipids, nucleic acids, organic acids, carbohydrates, biogenic amines, minerals, vitamins, food additives, drugs, cosmetics, contaminants, pollutants, and any chemical that humans assume or metabolize, or come into contact with. We used the Human Metabolome Database (HMDB; https://hmdb.ca/ (accessed on 28 February 2021)) to get the list of human metabolites to be used for analysis [22,43,44,45]. We restricted the search to molecules with molecular weight (M.W.) between 250 and 1000 Da for which a structure file was available. Further considerations, essentially based on the rule of five, were made for the selection of the molecules to be analyzed [46]. SMILES codes [47] of these molecules have been used in ZINC 15 to obtain files in *pdbqt* format [48]. Further data on the selected molecules were obtained from PubChem [49]. The structural analysis of M^pro^ was conducted, essentially as previously described [26,50,51,52] in a VMD (version 1.9.3) [53] environment. Atomic coordinates of the SARS-CoV-2 M^pro^ were obtained from the PDB [54] entry 5RET [29]. Molecular docking was performed using the AutoDock Vina software (version 1.1.2 linux_x86) [55]; *pdbqt* files were obtained the same software or by the Open Babel toolbox (version 2.3.2) [56]. Docking analyses were performed on the 5RET structure [29]. The protein target *pdbqt* files were obtained by adding hydrogen atoms and charges were assigned using the Gasteiger method, using AutoDock Tools (version 1.5.6) [57]. Docking boxes were centered on the sulfur atom of Cys-145. The box dimensions were (28 × 32 × 34) Å.

To test the stability of the M^pro^-ligand complex [58,59], molecular dynamics of the complex has been performed using NAMD [60], in a water box with periodic boundary conditions essentially as described [61]. The parameterization of the ligand was carried out by means of the CHARMM-GUI [62,63,64].

### 4.2. In Vitro Analyses

Enzymatic assays were performed essentially as described in our previous work [65]. Briefly, we used the purified SARS-CoV-2 M^pro^ Untagged purchased from BPS Bioscience (cat. no. 100823-1) (San Diego, CA, United State) at a final concentration value of 0.5 ng/µL in the reaction buffer supplied by the manufacturer (BPS Bioscience; Cat. No. 79956). Silymarin (Cat. No. HY-N7073) and taxifolin (cat. no. HY-N0136) were purchased from MedChemExpress (MCE) (Monmouth Junction, NJ 08852, USA). Experiments were performed at room temperature in a Tecan microplate reader using an internally quenched fluorogenic FRET substrate (DABCYL-KTSAVLQSGFRKME-EDANS) (BPS Bioscience; Cat. No. 79952-1) as substrate at a concentration value of 40 µM. For this peptide, a K_m_ of 17 µM and a K_cat_ of 1.9 s^−1^ on the M^pro^ have been reported. The experimental veterinary drug GC376 [19,20] (BPS Bioscience; Cat. No. 78013) was used at a concentration value of 100 µM as a positive control. The latter is capable of inhibiting SARS-CoV-2 M^pro^ with an IC50 of approximately 0.42 µM. The assays were carried out in the reaction buffer supplied by the manufacturer, in the presence of 0.1 µM of DTT derived from the storage solution of the enzyme (DTT free condition) or in the presence of 1 mM of DTT.

## Data Availability

Not applicable.

## Supplementary Materials

The following are available online. Table S1: The metabolites data set and binding energies on 5RET.
